# Characterization of the microbial communities in paddy soils in Lajas, Puerto Rico using 16S rRNA gene

**DOI:** 10.1128/mra.01008-24

**Published:** 2024-12-10

**Authors:** Edwin Omar Rivera-Lopez, Javier Huertas-Miranda, Carlos Rios-Velazquez

**Affiliations:** 1 Food Science and Technology Program, University of Puerto Rico at Mayagüez, Mayagüez, Puerto Rico, USA; 2 Microbial Biotechnology and Bioprospecting Laboratory, Biology Department, University of Puerto Rico at Mayagüez, Mayagüez, Puerto Rico, USA; DOE Joint Genome Institute, Berkeley, California, USA

**Keywords:** paddy soil, pre-harvest, post-harvest, soil microbiome, Puerto Rico

## Abstract

The microbiota in the paddy soils of the Lajas Agricultural Experimental Station at the University of Puerto Rico (LAES-UPR) plays a crucial role in agricultural ecosystems. Despite being at an experimental station, these soils represent natural environments supporting rice cultivation. Microbial diversity was evaluated during pre-harvest and post-harvest periods.

## ANNOUNCEMENT

Soil is a complex ecosystem where microorganisms interact, significantly contributing to soil quality and productivity (1). Paddy soils are unique due to their saturation with standing water, remaining flooded during growth and periodically draining during harvest (2). This creates three regions with different physicochemical characteristics: the aerobic surface, the anoxic layer, and the rhizosphere (3). Understanding microbial communities in paddy soils is essential for soil dynamics and agricultural productivity. Dominant phyla identified in these environments include Chloroflexi, Actinobacteria, Acidobacteria, Proteobacteria, Verrucomicrobia, and Bacteroidetes (4, 5).

Pre-harvest and post-harvest soil samples were collected from the aerobic surface (2–4 cm depth) at LAES-UPR (18.0156508,–67.0744860) in January 2019 and September 2020, respectively. Large particles were removed, followed by chemical, enzymatic, and mechanical DNA extraction. Two soil samples of 12.5 g were collected from each environment and combined with 20 mL of buffer-Z (100 mM Tris-HCl [pH 8.0], 100 mM sodium phosphate, 100 mM EDTA [pH 8.0], 1.5M NaCl, and 1% hexadecyltrimethylammonium bromide [CTAB]) and lysozyme (1 mg/mL). The mixture underwent a freeze-thaw cycle (40 min in liquid nitrogen, then 40 min at 65°C) and was centrifuged at 6240 × *g* for 10 min. The supernatant was transferred to a sterile 2 mL microtube and centrifuged at 16,873 × *g* for 20 min. Then, 1 mL of the supernatant was mixed with an equal volume of chloroform: isoamyl alcohol solution (24:1), inverted gently for 10 min, and centrifuged at 16,873 × *g* for 20 min. The supernatant was mixed with 0.7 volumes of cold isopropanol, inverted for 5 min, and incubated on ice for 10 min. DNA was precipitated by centrifugation at 16,873 × *g* for 40 min, and the supernatant was carefully removed. The microtubes were dried for 10 minutes to remove traces of isopropanol. The pellet was resuspended in 50 µL of Tris-EDTA (TE) buffer (10 mM Tris-HCl, 1 mM EDTA) using broad-tip microtips to avoid DNA fragmentation, following pooling of DNA from the pre-harvest and post-harvest extractions (6–11). The 16S rRNA gene was amplified using primers 515F (GTGYCAGCMGCCGCGGTAA) and 806R (GGACTACNVGGGTWTCTAAT) (12), and the metagenomic DNA was sent to Mr. DNA’s laboratory for sequencing. The sample was fragmented, adapter sequences were added, and sequencing was performed on the Illumina MiSeq system with paired-end reads over 600 cycles. Post-sequencing, samples were trimmed to 250 bp using Qiita (version 2022.11) and processed in Qiime (version 1.9.1) (13), resulting in 35,426 reads for microbial taxa analysis. The data were clustered into 1,375 OTUs at 97% similarity threshold, representing pre-harvest and post-harvest samples, and rarefied for equal sequencing depth.

**Fig 1:**
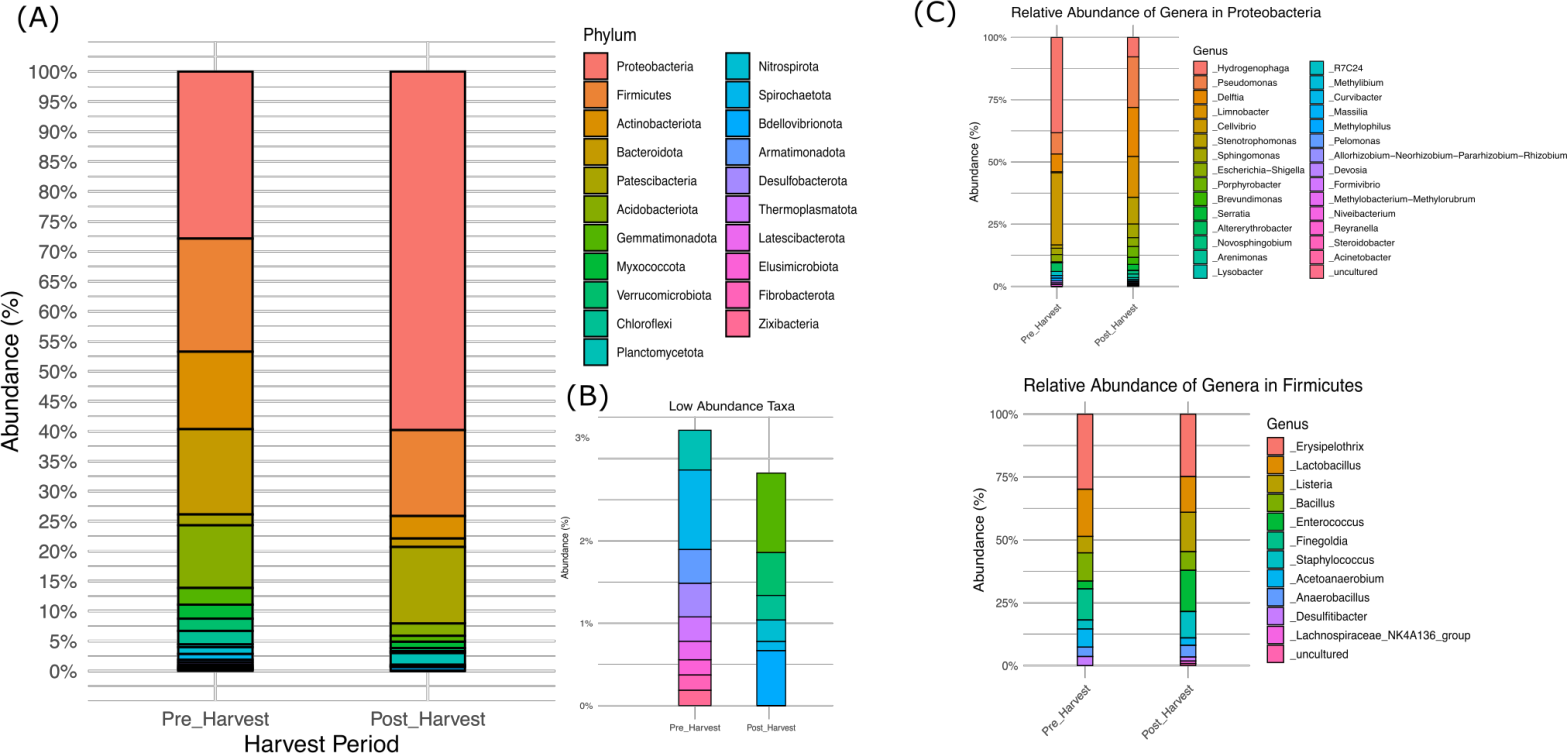
Community structures of the pre-harvest and post-harvest paddy soils. (**A**) Taxa bar plots show the relative abundance of bacteria at the phylum levels. (**B**) This panel highlights low-abundance phyla that may not be prominently visible in panel A as a result of their minimal representation. (**C**) Taxa bar plots show the relative abundance of bacteria genera in the two most abundance phyla (Proteobacteria and Firmicutes).

The most abundant phyla were Proteobacteria (27.86%), Firmicutes (18.87%), Bacterioidota (14.22%), and Actinobacteria (12.92%) pre-harvest; post-harvest, they were Proteobacteria (59.80%), Firmicutes (14.33%), and Patescibacteria (12.74%)(Fig. 1A). The dominant genus in the Proteobacteria phyla pre-harvest was *Hydrogenophaga* (9.43%). Post-harvest, the dominant genera were *Pseudomonas* (11.85%), *Delftia* (11.48%), and *Limnobacter* (9.51%) (Fig. 1C). In the Firmicutes phylum, Erysipelothrix and Lactobacillus were dominant in both environments. These results provide important information about microbial communities in the paddy soils from Lajas, Puerto Rico.

## Data Availability

This project has been deposited in the National Center for Biotechnology Information (NCBI) under the accession number (PRJNA1096947), with raw reads from both pre-harvest (barcode sequence CAGTTCAT) and post-harvest (barcode sequence CAGTTGCA) libraries available under the same SRA accession number (SRR28574492).

## References

[1] Gopinath SCB, Anbu P, Arshad MKM, Lakshmipriya T, Voon CH, Hashim U, Chinni SV. 2017. Biotechnological processes in microbial amylase production. Biomed Res Int 2017:1272193. doi:10.1155/2017/1272193 28280725 PMC5322433

[2] Lopes AR, Faria C, Prieto-Fernández Á, Trasar-Cepeda C, Manaia CM, Nunes OC. 2011. Comparative study of the microbial diversity of bulk paddy soil of two rice fields subjected to organic and conventional farming. Soil Biol Biochem 43:115–125. doi:10.1016/j.soilbio.2010.09.021

[3] Witt C, Haefele SM. 2005. Paddy soils,p 141–150. In Hillel D (ed), Encyclopedia of soils in the environment. Elsevier. ISBN 9780123485304.

[4] Liesack W, Schnell S, Revsbech NP. 2000. Microbiology of flooded rice paddies. FEMS Microbiol Rev 24:625–645. doi:10.1111/j.1574-6976.2000.tb00563.x 11077155

[5] Jiang H, Dong H, Zhang G, Yu B, Chapman LR, Fields MW. 2006. Microbial diversity in water and sediment of Lake Chaka, an athalassohaline lake in northwestern China. Appl Environ Microbiol 72:3832–3845. doi:10.1128/AEM.02869-05 16751487 PMC1489620

[6] Cruz JM, Ortega MA, Cruz JC, Ondina P, Santiago R. 2010. Unraveling activities by functional-based approaches using metagenomic libraries from dry and rain forest soils in Puerto Rico. Current research, technology and education topics in applied microbiology and microbial biotechnology 2:1471–1478.

[7] Liles MR, Williamson LL, Handelsman J, Goodman RM. 2004. Isolation of high molecular weight genomic DNA from soil bacterial for genomic library construction.

[8] Miller DN, Bryant JE, Madsen EL, Ghiorse WC. 1999. Evaluation and optimization of DNA extraction and purification procedures for soil and sediment samples. Appl Environ Microbiol 65:4715–4724. doi:10.1128/AEM.65.11.4715-4724.1999 10543776 PMC91634

[9] Steffan RJ, Goksøyr J, Bej AK, Atlas RM. 1988. Recovery of DNA from soils and sediments. Appl Environ Microbiol 54:2908–2915. doi:10.1128/aem.54.12.2908-2915.1988 2851961 PMC204403

[10] Zhou J, Bruns MA, Tiedje JM. 1996. DNA recovery from soils of diverse composition. Appl Environ Microbiol 62:316–322. doi:10.1128/aem.62.2.316-322.1996 8593035 PMC167800

[11] Krsek M, Wellington EM. 1999. Comparison of different methods for the isolation and purification of total community DNA from soil. J Microbiol Methods 39:1–16. doi:10.1016/s0167-7012(99)00093-7 10579502

[12] Merkel AY, Tarnovetskii IY, Podosokorskaya OA, Toshchakov SV. 2019. Analysis of 16S rRNA primer systems for profiling of thermophilic microbial communities. Microbiology (Reading, Engl) 88:671–680. doi:10.1134/S0026261719060110

[13] Rodríguez-Barreras R, Tosado-Rodríguez EL, Godoy-Vitorino F. 2021. Trophic niches reflect compositional differences in microbiota among Caribbean sea urchins. PeerJ 9:e12084. doi:10.7717/peerj.12084 34540373 PMC8415288

